# CT-guided infiltration of the ischiofemoral space in young patients with ischiofemoral impingement is an effective diagnostic tool

**DOI:** 10.1186/s13244-024-01815-4

**Published:** 2024-10-07

**Authors:** Alexander F. Heimann, Moritz Wagner, Peter Vavron, Alexander Brunner, Ricardo Donners, Ehrenfried Schmaranzer, Simon D. Steppacher, Moritz Tannast, Reto Sutter, Florian Schmaranzer

**Affiliations:** 1 Department of Orthopaedic Surgery, HFR—Cantonal Hospital, University of Fribourg, Fribourg, Switzerland; 2 Center for Computer Assisted & Reconstructive Surgery, New England Baptist Hospital, Boston, MA USA; 3 Department of Orthopaedic Surgery, District Hospital St. Johann in Tirol, St. Johann in Tirol, Austria; 4 Department of Radiology, University Hospital Basel, Basel, Switzerland; 5 Department of Radiology, District Hospital St. Johann in Tyrol, St. Johann in Tirol, Austria; 6 Department of Orthopaedic Surgery, Inselspital Bern, University Hospital, University of Bern, Bern, Switzerland; 7 Department of Radiology, Balgrist University Hospital, Zurich, Switzerland; 8 Department of Diagnostic, Interventional and Pediatric Radiology, Inselspital, Bern University Hospital, University of Bern, Bern, Switzerland

**Keywords:** Hip, Femoroacetabular impingement, Ischiofemoral impingement, Diagnostic injection, Femoral torsion

## Abstract

**Objectives:**

To present our technique of diagnostic CT-guided ischiofemoral space injection and report on pain response, complications, and associated imaging findings in young patients with ischiofemoral impingement (IFI).

**Methods:**

Retrospective case series of patients with a clinical diagnosis of IFI that underwent CT-guided IFS injection with local anesthetic in a prone position with the feet in maximum internal rotation between 06/2019 and 04/2021. The response was evaluated using maximum subjective pain evaluation on a 0–10 visual analog scale (VAS) during a standardized pre- and postinterventional clinical examination. Patient charts and radiographic imaging data were reviewed to report associated imaging findings and subsequent surgeries.

**Results:**

Eleven patients (13 hips, 9 females) with a median age of 31 years (interquartile range; IQR: 25–37 years) were included. Median baseline VAS was 7 points (IQR: 5–8) with a pain reduction of 5 points (IQR: 5–7 points, *p* = 0.001) after the injection. One patient reported transient ischial nerve paresthesia, otherwise, no complications occurred. Quadratus femoris muscle edema was present in 85% (11 of 13 hips). Excessively high femoral torsion (11/13 hips, 85%) and cam deformities (8/13 hips, 62%) were the most common osseous deformities. Eight of 13 hips (62%) underwent subsequent surgery for IFI.

**Conclusion:**

CT-guided diagnostic injection of the ischiofemoral space is safe and feasible. In young IFI patients, diagnostic IFS injections have the potential to improve the differential diagnosis of hip pain and to inform decision-making with regard to a possible benefit of joint-preserving hip surgery.

**Critical relevance statement:**

In young patients with hip pain, diagnosis of IFI can be challenging due to concomitant pathologies. Furthermore, surgical treatment in these patients is controversial. In this context, CT-guided diagnostic infiltrations of the ischiofemoral space may facilitate not only the initial diagnosis of IFI, but could also improve surgical decision-making.

**Key Points:**

- CT-guided diagnostic injection of local anesthetic in the ischiofemoral space is safe.
- In young patients with IFI, it leads to subjective pain reduction.
- In young patients with concomitant osseous deformities, it may improve surgical decision-making.

**Graphical Abstract:**

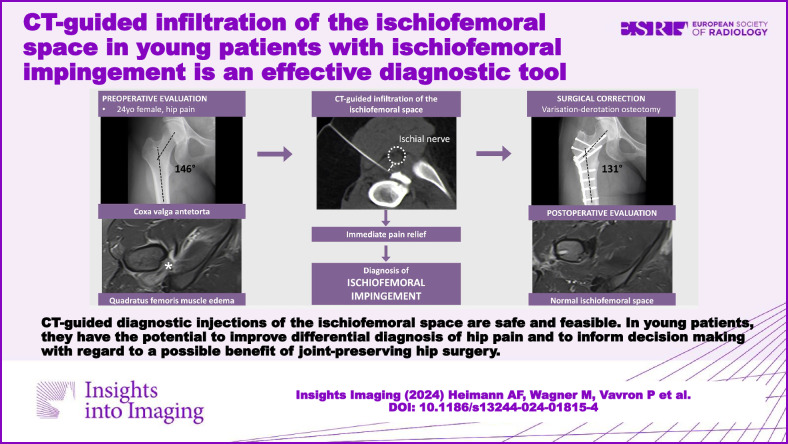

## Introduction

Ischiofemoral impingement (IFI) is more common in women and often occurs bilaterally [1, 2]. The underlying abnormal narrowing of the space between the ischium and the posterior part of the proximal femur not only causes symptoms in older patients, but has also been identified as a cause of hip pain in young patients [3]. Patients with IFI usually complain of pain in the hip and/or buttocks. The clinical diagnosis is based on the reproduction of the known pain during movement patterns that lead to a further narrowing of the already reduced ischiofemoral space (IFS). This includes long stride-walking [4], a combined flexion–abduction–external rotation movement (FABER test) [5, 6], or during combined extension–adduction–external rotation (posterior impingement test). However, pain during normal gait or at rest accompanied by a sensation of clicking or snapping may also occur [7, 8]. The etiology of IFI is multifactorial and implicates abnormal shape of the pelvis and ischium [2, 9], femoral malalignment [10–12], as well as functional factors such as muscular dysbalance [13, 14]. Imaging diagnosis of IFI is based on static or dynamic visualization of the narrowing of the IFS in combination with a quadratus femoris muscle (QFM) edema on magnetic resonance imaging (MRI) with fluid-sensitive sequences [2, 6, 15, 16]. Treatment strategies for IFI depend on the underlying etiology and on the patient's age [17]. In young patients with femoral malalignment due to excessively high femoral torsion, femoral derotational osteotomies or resection of the lesser trochanter have been introduced for surgical treatment of IFI. However, in practice, it is often difficult to establish a clinical diagnosis of IFI [17, 18].

Recently corticoid and local anesthetic injections into the IFS have been proposed as a diagnostic [19], or even therapeutic tool [17, 20]. To date, this is yet to be applied in young patients who are eligible for joint-preserving hip surgery. Thus the aim of this study was to describe our technique of diagnostic computed tomography (CT)-guided injection of the IFS. In addition, we report on the pain response, complications, and imaging findings in young IFI patients undergoing evaluation for surgical correction of IFI.

## Materials and methods

### Patients

Institutional Review Board (IRB) approved a single-center, retrospective observational study conducted at a primary referral center for joint preserving hip surgery in Austria. The study was performed with a written informed consent waiver. We included all patients with a clinical diagnosis of IFI who underwent CT-guided injection of local anesthetic into the IFS at our institution between June 2019 and April 2021. The overall study cohort was 13 hips (11 patients). Patient records were studied for subsequent surgeries.

### Clinical diagnosis of IFI

**Fig. 1:**
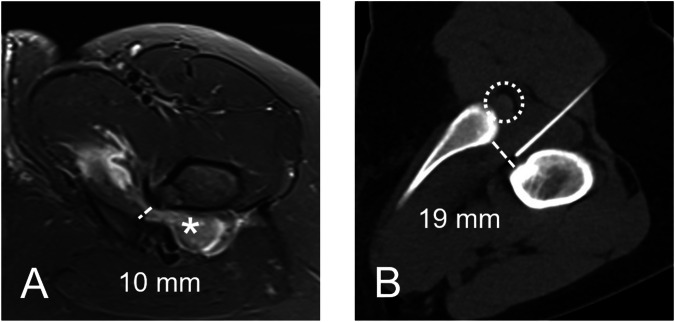
**A** Axial short-tau inversion recovery MR-image with a narrow IFS of 10 mm (dashed line) and concomitant QFM edema (asterisk) in the supine position and neutral leg rotation. **B** Axial CT-image during diagnostic IFS infiltration in the prone position and maximum internal rotation, allowing enlargement of the IFS to 19 mm (dashed line) and sufficient distance of the needle to the ischial nerve (dotted circle) during injection of the local anesthetic

Diagnosis of IFI was primarily based on the clinical presentation. This was a typical history of pain in conjunction with pain during clinical examination, and a positive FABER and/or posterior impingement test [18]. The suspected clinical diagnosis of IFI was subsequently confirmed by IFI-typical morphology (narrowing of the IFS or QFM edema, Fig. 1A) and the absence of other causes of secondary IFI on diagnostic imaging such as tumors or tumor-like lesions [2].

### Diagnostic imaging

All patients underwent diagnostic imaging of the symptomatic hip including supine anteroposterior pelvis radiographs and 45° modified Dunn-views [21]. Affected hips underwent direct MR arthrography at 1.5 T (Magnetom Aera, Siemens Healthineers) following fluoroscopically guided intra-articular injection of 15–20 mL of diluted MR contrast agent (gadopentetate dimeglumine, 2.0 mmol/L; Magnevist; Bayer Healthcare) or 15–20 mL 0.9% NaCl solution (0.9% NaCl Fresenius; Fresenius Kabi) [22], 1–2 mL iodinated contrast agent (iopamidol, 200 mg/mL; Iopamiro 200; Bracco) and injection of 2–5 mL local anesthetic (ropivacaine hydrochloride; 2 mg/mL; Ropinaest; Gebro Pharma).

Our institutional protocol includes an axial short-tau inversion recovery sequence of the pelvis with a repetition time/echo time of 4360/42 ms, matrix of 384 × 384, field of view of 360 mm, flip angle of 150°, slice thickness of 5 mm and bandwidth of 161 Hz/Px. For measurement of femoral torsion 3D T1-weighted volumetric interpolated breath-hold examination DIXON sequences of the pelvis and distal femoral condyles were acquired: repetition time/echo times of 6.7/2.4 and 4.8 ms, matrix of 320 × 320, field of view of 380 mm, flip angle of 10°, slice thickness of 3 mm and bandwidth of 470 Hz/Px. Additionally, proton-density weighted turbo spin echo images without fat saturation were acquired with weight-adjusted lower limb traction [22, 23] in coronal, axial-oblique, sagittal, and radial orientation: repetition time/echo time of 2460/13 ms, matrix of 512 × 512, field of view of 180 mm, flip angle of 150°, slice thickness of 3 mm and bandwidth of 130 Hz/Px.

### CT-guided infiltration of the IFS

All patients underwent CT-guided injection of local anesthetic into the IFS in an outpatient setting. Prior to the injection, patients underwent clinical examination by an orthopedic surgeon. After performing both a FABER test and a posterior impingement test, patients were asked to report their maximum pain on a visual analog scale (VAS), on which zero points equal no pain and ten points equal the strongest pain imaginable. To perform the CT-guided injection, the patients were placed in the prone position with legs fixed in maximum internal rotation with a tape to widen the IFS as much as possible to ensure safe needle passage at the greatest possible distance from the sciatic nerve. Using CT-guidance in the axial plane, the IFS dimension was measured as the shortest distance between the lateral cortex of the ischial tuberosity and the medial cortex of the lesser trochanter [3] (Fig. 1B). We used a posterolateral approach, as described by Hernando et al [8], where the entry point of the needle was located on the posterolateral part of the buttocks. Using a 22-gauge needle, the trajectory was then pointed in a direct line to the center of the QFM. During subsequent needle insertion, special attention was paid to keeping the tip of the needle close to the lesser trochanter in order not to harm the sciatic nerve. The needle was advanced into the center of the QFM and correct positioning of the tip was controlled in all patients using 1 mL of iodinated contrast agent (iopamidol, 200 mg/mL; Iopamiro 200; Bracco). Then, an injection of 3–5 mL of local anesthetic (ropivacaine hydrochloride; 2 mg/mL; Ropinaest; Gebro Pharma) was carried out. Finally, the needle was removed, and the patients were monitored for a short time, during which the function of the sciatic nerve was checked. Thirty minutes after the injection, the same orthopedic surgeon re-examined the patients and re-performed a FABER and posterior impingement test. Patients were again asked to report their maximum pain during examination using the VAS.

### Image analysis

All measurements were performed by a fellowship-trained musculoskeletal radiologist with 8 years of hip imaging experience (F.S.). Morphological hip assessment was performed according to our institutional standard for patients being evaluated for joint preserving hip surgery, following the recommendation by the Lisbon agreement on femoroacetabular impingement (FAI) assessment [24]. Hence, an assessment of acetabular coverage (lateral center edge angle), signs of acetabular retroversion (cross-over, posterior wall, and ischial spine sign), and measurement of femoral neck-shaft angle were carried out. Femoral torsion was measured on MRI according to Murphy et al [25, 26] and excessive femoral torsion was defined as > 35°. Acetabular version measurement was performed at the midlevel of the femoral head [27]. IFS was measured on MRI as the shortest distance between the lateral cortex of the ischial tuberosity and the medial cortex of the lesser trochanter [3]. The ischial angle was measured as described by Bredella et al [2] and QFM edema was considered to be present, if increased signal intensity in the QFM on axial short-tau inversion recovery images was seen (Fig. 1A). On the interventional CT, IFS with the legs fixed in maximal internal rotation was measured. The shortest distance (in mm) of the needle trajectory to the ischial nerve was measured in all patients. The presence of cartilage damage and labral lesions (labral base tear, intrasubstance tears, and complex tears) in the hip joint was assessed [28].

### Statistical analysis

Statistical analysis was performed using MedCalc (MedCalc Statistical Software, version 20.106, MedCalc Software Ltd, Ostend, Belgium). Due to the small sample size only non-parametric statistics were used and numerical variables were reported using the median (interquartile range [IQR]). To evaluate the influence of maximal internal rotation on the width of the IFS, the absolute width of the IFS in millimeters (mm) was compared between neutral rotation on MRI and maximal internal rotation on CT using the Wilcoxon test. Analogously, a comparison of the pre- and postinterventional VAS was performed using a Wilcoxon test. Comparison of the pre- and postinterventional VAS allowed for the calculation of median absolute pain reduction.

## Results

### Patient characteristics

The study group consisted of 13 hips of 11 patients (81% female) with a median age of 31 years (IQR: 25–37 years, Table 1). Eight of 13 hips (62%) underwent subsequent surgery. Of these, four hips (50%) underwent surgical hip dislocation with either concomitant intertrochanteric variation-derotational femoral osteotomy (two hips), or partial reduction of the lesser trochanter (two hips). In two hips (25%) hip arthroscopy with concomitant subtrochanteric derotational femoral osteotomy was performed. One (13%) hip underwent isolated subtrochanteric femoral derotational osteotomy and one hip (13%) underwent isolated periacetabular osteotomy. Femoral osteochondroplasty with improvement of the head-neck offset was performed in five hips (63%).

**Table 1:** Patient demographics and osseous deformities

Parameter	Study group (13 hips)
Age (y), median (IQR)	31 (25–37)
Female sex	9
Bilateral	2
Osseous deformities (number of hips)	
Excessively high femoral torsion (> 35°)	11
Valgus deformity (neck shaft angle > 135°)	5
Cam deformity (α angle > 60°)	8
Hip dysplasia (LCE < 25°)	1
Acetabular over coverage (LCE > 35°)	1
Acetabular retroversion^*^	2

If not otherwise noted, values are depicted as *n*

^*^ Defined as all three retroversion signs positive

### CT-guided IFI infiltration: response and complications

Median baseline VAS was 7 points (IQR: 5–8) with a pain reduction of 5 points (IQR: 5–7 points, *p* = 0.001) reported by all patients (13 of 13 hips) 30 min after the infiltration.

Compared to the neutral rotation in the supine position measured on MRI (median 17 mm, IQR: 15–19 mm), the prone position with maximal hip internal rotation (median 22 mm, 20–26 mm) allowed for significant widening of the IFS (median widening of 5 mm, IQR: 2–9 mm, *p* = 0.002). The median distance between the needle and the ischial nerve in the IFS was 6 mm (IQR: 3–9 mm).

One patient (one hip) complained of paresthesia of the ipsilateral sciatic nerve in the first 30 min following the infiltration, which resolved completely. No other complications were observed.

### Associated imaging findings

Median femoral torsion was 49° (IQR: 49°–53°). Excessively high femoral torsion (> 35°) was present in 11 of 13 hips (85%). The median neck shaft angle was 134° (IQR: 135°–139°) and 5 of 13 hips (38%) had a valgus deformity (> 135°). Eight of 13 hips (62%) had additional osseous deformities: cam deformities present in 8 of 13 hips (62%), pincer deformities present in 3 of 13 (23%) hips, and one hip (8%) had acetabular dysplasia. MR-imaging revealed a QFM edema in 85% (11 of 13 hips). All hips showed labral tears, most of which were intrasubstance- or complex tears (9 of 13 hips, 69%). An overview of radiographic and MRI findings is shown in Table 2.

**Table 2:** Radiographic and MR-arthrography imaging findings

Parameter	11 patients (13 hips)
QFM edema, (number of hips, %)	11 (85)
IFS in neutral position, (mm)	17 (15–19)
IFS in internal rotation, (mm)	22 (20–26)
Ischial angle, (°)	133 (131–137)
Femoral torsion, (°)	49 (49–53)
High femoral torsion > 35°, (number of hips, %)	11 (85)
Neck shaft angle (°)	134 (130–139)
Valgus deformity > 135°, (number of hips, %)	5 (38)
α angle, (°)	65 (55–71)
Cam deformity > 60°, (number of hips, %)	8 (62)
Central acetabular version, (°)	22 (18–25)
Lateral center edge-angle, (°)	29 (28–32)
Hip dysplasia < 25°, (number of hips, %)	1 (8)
Overcoverage > 35°, (number of hips, %)	1 (8)
Acetabular retroversion, (number of hips, %)	2 (15)
Cross-over sign	3 (23)
Ischial spine sign	2 (15)
Posterior wall sign	3 (23)
Labral tear, (number of hips, %)	13 (100)
Intrasubstance or complex tears	9 (69)
Labral base tear	4 (31)
Cartilage damage, (number of hips, %)	10 (77)

Numerical values are depicted as median (IQR). Categorical values are depicted as *n* (%)

## Discussion

In this retrospective case series, we describe our experience with CT-guided injection of the IFS using a posterolateral approach in young patients with a clinical diagnosis of IFI and report associated imaging findings. Despite the multifactorial etiology, the pathomechanism of IFI always involves a dynamic narrowing of the IFS. This narrowing of the IFS leads to impingement of the neuromuscular structures within the IFS, particularly the QFM, and the resulting myofascial pain is considered to be the major contributor to IFI symptoms [13, 20]. Injections of local anesthetics and corticosteroids are low-risk, cost-effective procedures for a variety of musculoskeletal disorders, both for diagnostic purposes and for short- and mid-term pain management. Recently, these injections have also been proposed as part of the treatment algorithm for IFI patients [17]. In our institution, we further modified the CT-guided posterolateral approach with the patient in the prone position, as proposed by Hernando et al [8], by fixing the patient’s legs in maximum internal rotation to widen the ischiofemoral interval for a safe needle passage. This technique allowed for excellent visualization of the anatomical landmarks, IFS, QFM, as well as the ischial nerve. Using our technique, we observed a substantial median pain reduction of 5 points (IQR: 5–7) in all patients, which was combined with the absence of relevant complications in our study. This is consistent with the pain relief and safety of CT-guided IFS infiltrations previously reported in several case reports [19, 29, 30], and a case series [20].

**Fig. 2:**
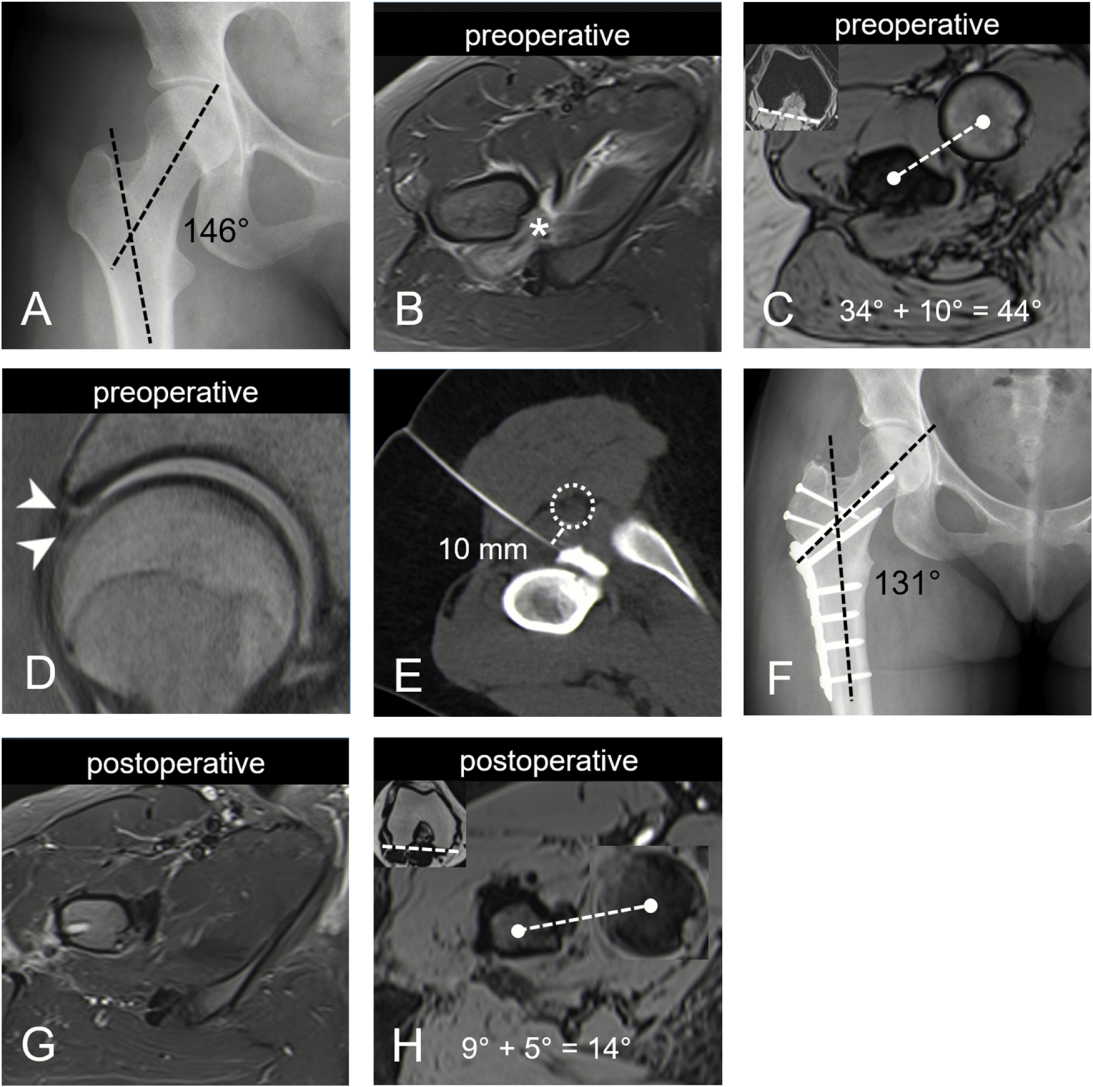
Anteroposterior pelvic radiograph of a 24-year-old female patient with right buttock pain and positive posterior impingement test on clinical examination. Diagnostic imaging revealed (**A**) a valgus deformity of the right hip on radiographs, (**B**) narrowing of the IFS with concomitant QFM edema (asterisk) on a preoperative axial short-tau inversion recovery MR-image, (**C**) increased femoral torsion (44°) measured on axial T1-w volume-interpolated breath-hold examination DIXON MR-image, as well as a (**D**) concomitant intrasubstance tear of the anterosuperior labrum (arrowheads) on a sagittal proton-density weighted turbo spin echo MR-image. This led to the clinical suspicion of IFI. The patient underwent (**E**) CT-guided diagnostic infiltration of the IFS in the prone position from a posterolateral approach with immediate post-procedural pain relief. The distance between the needle trajectory and the ischial nerve (dotted circle) was 10 mm (dashed line). Subsequently, (**F**) the patient underwent surgical hip dislocation with additional intertrochanteric varisation-derotation osteotomy with relief of symptoms. Postoperative MR-imaging following hardware removal performed for evaluation of the contralateral hip revealed a (**G**) normalization of the IFS and (**G**) corrected femoral torsion (14°, **H**)

Compared to the recently published case series of 12 patients (median age 58 years) who underwent CT-guided IFS infiltration [20], our cohort was significantly younger, with a median age of 31 years. Hence, the patients suffering from hip pain in our series were all undergoing diagnostic evaluation for the potential benefit of joint-preserving hip surgery. Unlike previous studies which did not report on associated osseous deformities and imaging findings, our patients showed several typical findings of IFI. This included a high prevalence of QFM edema (85%) [2, 15] in combination with a narrow IFS [2, 3, 30], increased femoral torsion (85%) [6, 31], or valgus deformity (38%) [10]. This is consistent with clinical studies reporting on patients undergoing femoral derotational osteotomies for the treatment of IFI [17, 18]. Interestingly, there was also a high prevalence of intrasubstance or complex labral tears (69%) in the hip joints of these young patients with IFI. This pattern of labrum damage has recently been associated with posterior extra-articular impingement with anterior subluxation of the femoral head in patients with excessive femoral torsion [32]. However, in addition to these IFI-typical findings, many patients (62%) also showed a cam deformity, while one patient had hip dysplasia and three others had a pincer deformity. This illustrates the potential value of diagnostic infiltrations in young IFI patients who also have other associated osseous deformities that are commonly linked with classic FAI that could explain their symptoms. Similarly to intra-articular injections performed in patients with intra-articular FAI [33, 34], a positive response to diagnostic infiltration of the IFS may inform the potential benefit of surgical treatment. Accordingly, 62% of hips in our study underwent subsequent surgery, with either a femoral osteotomy or partial resection of the lesser trochanter being performed during seven of the eight procedures carried out. The direct aim of both procedures was to mechanically widen the narrowed IFS and ensure impingement-free mobility, as demonstrated in the clinical case presented in Fig. 2.

This study had limitations. First, this is a single-center, retrospective case series with a small sample size, reducing the generalizability of our findings. As a result, the findings might not be fully applicable to other settings or populations. Second, the radiologic diagnosis of IFI was made by a single investigator, which introduces a potential observer bias and limits the ability to assess the consistency of the diagnosis. Third, our study only provides immediate post-procedural outcomes, and pain response was assessed using a VAS, which could introduce variability based on individual pain tolerance and perception. Long-term effects and potential recurrence of symptoms were not evaluated. Hence, the potential influence of the result of diagnostic IFS infiltration on surgical decision-making cannot be quantified based on this retrospective analysis. Fourth, therapeutic infiltrations with corticosteroids were not performed, so no conclusions about their potential value can be drawn based on our results.

In this case series, we report on our technique of CT-guided diagnostic injection of the IFS and demonstrate its safety and feasibility. Especially in young patients with IFI and combined pathomorphologies, these injections can help in the differential diagnosis of hip pain and can support surgical decision-making.

## Data Availability

The datasets used and/or analyzed during the current study are available from the corresponding author upon reasonable request.

## Abbreviations

CT: Computed tomography
FABER: Flexion–abduction–external rotation
FAI: Femoroacetabular impingement
IFI: Ischiofemoral impingement
IFS: Ischiofemoral space
IQR: Interquartile range
MRI: Magnetic resonance imaging
VAS: Visual analog scale

## References

[1] Taneja A, Bredella M, Torriani M (2013) Ischiofemoral impingement. Magn Reson Imaging Clin N Am 21:65–73. 10.1016/j.mric.2012.08.005 23168183 10.1016/j.mric.2012.08.005

[2] Bredella M, Azevedo D, Oliveira A et al (2015) Pelvic morphology in ischiofemoral impingement. Skeletal Radiol 44:249–253. 10.1007/s00256-014-2041-0 25371087 10.1007/s00256-014-2041-0

[3] Torriani M, Souto S, Thomas B et al (2009) Ischiofemoral impingement syndrome: an entity with hip pain and abnormalities of the quadratus femoris muscle. AJR Am J Roentgenol 193:186–190. 10.2214/AJR.08.2090 19542413 10.2214/AJR.08.2090

[4] Li Y-P, Li G-P, Liu K et al (2022) Interpretation of ischiofemoral impingement via a clinical test using hip triaxial dynamic magnetic resonance imaging. Quant Imaging Med Surg 12:384–394. 10.21037/qims-21-292 34993087 10.21037/qims-21-292 PMC8666745

[5] Bagwell J, Bauer L, Gradoz M, Grindstaff T (2016) THE RELIABILITY OF FABER TEST HIP RANGE OF MOTION MEASUREMENTS. Int J Sports Phys Ther 11:1101–1105 27999724 PMC5159634

[6] Heimann AF, Walther J, Tannast M et al (2023) Hip MRI in flexion abduction external rotation for assessment of the ischiofemoral interval in patients with hip pain—a feasibility study. Insights Imaging 14:172. 10.1186/s13244-023-01524-4 37840102 10.1186/s13244-023-01524-4 PMC10577115

[7] Patti J, Ouellette H, Bredella M, Torriani M (2008) Impingement of lesser trochanter on ischium as a potential cause for hip pain. Skeletal Radiol 37:939–941. 10.1007/s00256-008-0551-3 18682931 10.1007/s00256-008-0551-3

[8] Hernando M, Cerezal L, Pérez-Carro L et al (2016) Evaluation and management of ischiofemoral impingement: a pathophysiologic, radiologic, and therapeutic approach to a complex diagnosis. Skeletal Radiol 45:771–787. 10.1007/s00256-016-2354-2 26940209 10.1007/s00256-016-2354-2

[9] Audenaert E, Duquesne K, De Roeck J et al (2021) Ischiofemoral impingement: the evolutionary cost of pelvic obstetric adaptation. J Hip Preserv Surg 7:677–687. 10.1093/jhps/hnab004 34548927 10.1093/jhps/hnab004 PMC8448428

[10] Siebenrock K, Steppacher S, Haefeli P et al (2013) Valgus hip with high antetorsion causes pain through posterior extraarticular FAI. Clin Orthop Relat Res 471:3774–3780. 10.1007/s11999-013-2895-9 23463288 10.1007/s11999-013-2895-9 PMC3825876

[11] Morris W, Fowers C, Weinberg D et al (2019) Hip morphology predicts posterior hip impingement in a cadaveric model. Hip Int 29:322–327. 10.1177/1120700018779906 29808721 10.1177/1120700018779906

[12] Gardner S, Dong D, Peterson L et al (2020) Is there a relationship between femoral neck-shaft angle and ischiofemoral impingement in patients with hip pain? J Hip Preserv Surg 7:43–48. 10.1093/jhps/hnaa006 32382428 10.1093/jhps/hnaa006 PMC7195935

[13] Torriani M (2023) Editorial comment: CT-guided quadratus femoris injection for ischiofemoral impingement. Eur Radiol 33:3954–3955. 10.1007/s00330-023-09567-3 36977854 10.1007/s00330-023-09567-3

[14] Kheterpal A, Harvey J, Husseini J et al (2020) Hip abductor tears in ischiofemoral impingement. Skeletal Radiol 49:1747–1752. 10.1007/s00256-020-03497-7 32514583 10.1007/s00256-020-03497-7

[15] Singer A, Subhawong T, Jose J et al (2015) Ischiofemoral impingement syndrome: a meta-analysis. Skeletal Radiol 44:831–837. 10.1007/s00256-015-2111-y 25672947 10.1007/s00256-015-2111-y

[16] Vicentini J, Martinez-Salazar E, Simeone F et al (2021) Kinematic MRI of ischiofemoral impingement. Skeletal Radiol 50:97–106. 10.1007/s00256-020-03519-4 32638058 10.1007/s00256-020-03519-4

[17] Gollwitzer H, Banke I, Schauwecker J et al (2017) How to address ischiofemoral impingement? Treatment algorithm and review of the literature. J Hip Preserv Surg 4:289–298. 10.1093/jhps/hnx035 29250337 10.1093/jhps/hnx035 PMC5721376

[18] Lerch T, Schmaranzer F, Steppacher S et al (2022) Most of patients with femoral derotation osteotomy for posterior extraarticular hip impingement and high femoral version would do surgery again. Hip Int 32:253–264. 10.1177/1120700020953100 32866044 10.1177/1120700020953100

[19] Ali A, Whitwell D, Ostlere S (2011) Case report: imaging and surgical treatment of a snapping hip due to ischiofemoral impingement. Skeletal Radiol 40:653–656. 10.1007/s00256-010-1085-z 21207021 10.1007/s00256-010-1085-z

[20] Liou H, Long J, Kransdorf M, Schmieder S (2023) CT-guided quadratus femoris injection for ischiofemoral impingement. Eur Radiol 33:3956–3960. 10.1007/s00330-023-09497-0 36917261 10.1007/s00330-023-09497-0

[21] Tannast M, Siebenrock K, Anderson S (2007) Femoroacetabular impingement: radiographic diagnosis—what the radiologist should know. AJR Am J Roentgenol 188:1540–1552. 10.2214/AJR.06.0921 17515374 10.2214/AJR.06.0921

[22] Meier M, Wagner M, Brunner A et al (2023) Can gadolinium contrast agents be replaced with saline for direct MR arthrography of the hip? A pilot study with arthroscopic comparison. Eur Radiol. 10.1007/s00330-023-09586-0 10.1007/s00330-023-09586-0 PMC10415454 37042981

[23] Schmaranzer F, Klauser A, Kogler M et al (2015) Diagnostic performance of direct traction MR arthrography of the hip: detection of chondral and labral lesions with arthroscopic comparison. Eur Radiol 25:1721–1730. 10.1007/s00330-014-3534-x 25465714 10.1007/s00330-014-3534-x

[24] Mascarenhas V, Castro M, Rego P et al (2020) The Lisbon agreement on femoroacetabular impingement imaging-part 1: overview. Eur Radiol 30:5281–5297. 10.1007/s00330-020-06822-9 32405754 10.1007/s00330-020-06822-9

[25] Murphy S, Simon S, Kijewski P et al (1987) Femoral anteversion. J Bone Joint Surg Am 69:1169–1176 3667647

[26] Schmaranzer F, Kallini J, Miller P et al (2020) The effect of modality and landmark selection on MRI and CT femoral torsion angles. Radiology 296:381–390. 10.1148/radiol.2020192723 32515680 10.1148/radiol.2020192723

[27] Lerch T, Todorski I, Steppacher S et al (2018) Prevalence of femoral and acetabular version abnormalities in patients with symptomatic hip disease: a controlled study of 538 hips. Am J Sports Med 46:122–134. 10.1177/0363546517726983 28937786 10.1177/0363546517726983

[28] Lerch T, Nanavati A, Heimann A et al (2023) Are degenerative findings detected on traction MR arthrography of the hip associated with failure of arthroscopic femoroacetabular impingement surgery? Eur Radiol. 10.1007/s00330-023-10419-3 10.1007/s00330-023-10419-3 PMC11166863 37982837

[29] Volokhina Y, Dang D (2013) Using proximal hamstring tendons as a landmark for ultrasound- and CT-guided injections of ischiofemoral impingement. Radiol Case Rep 8:789. 10.2484/rcr.v8i1.789 27330616 10.2484/rcr.v8i1.789 PMC4900207

[30] Ali A, Teh J, Whitwell D, Ostlere S (2013) Ischiofemoral impingement: a retrospective analysis of cases in a specialist orthopaedic centre over a four-year period. Hip Int 23:263–268. 10.5301/hipint.5000021 23760746 10.5301/hipint.5000021

[31] Lerch T, Zwingelstein S, Schmaranzer F et al (2021) Posterior extra-articular ischiofemoral impingement can be caused by the lesser and greater trochanter in patients with increased femoral version: dynamic 3D CT-based hip impingement simulation of a modified FABER test. Orthop J Sports Med 9:2325967121990629. 10.1177/2325967121990629 34104657 10.1177/2325967121990629 PMC8167016

[32] Heimann AF, Todorski I, Schmaranzer F et al (2024) What is the influence of femoral version on size, tear location, and tear pattern of the acetabular labrum in patients with FAI? Clin Orthop Relat Res. 10.1097/CORR.0000000000002961 10.1097/CORR.0000000000002961 PMC11343535 38231022

[33] Takla A, Gunatilake K, Ma N, Moaveni A (2024) Can intra-articular hip injections predict arthroscopy outcomes for femoroacetabular impingement syndrome? A systematic review. J Orthop 50:122–129. 10.1016/j.jor.2023.12.004 38214002 10.1016/j.jor.2023.12.004 PMC10776375

[34] Martins E, Gomes D, de Brito Fontana H, Fernandes D (2023) Does response to preoperative intra-articular anesthetic injections predict outcomes of femoroacetabular impingement syndrome? Arch Orthop Trauma Surg 143:6283–6294. 10.1007/s00402-023-04927-6 37316693 10.1007/s00402-023-04927-6

